# Deciphering the toxicological mechanism of airway allergic diseases caused by dioctyl terephthalate: a composite study

**DOI:** 10.3389/ftox.2026.1743420

**Published:** 2026-03-11

**Authors:** Zhi-Qiang Zhang, You-Wei Bao, Hongyou Wang, Jing-Yang Li, Heng Wang, Xinyue Yang, Qi Chen, Jun Wu, Xin-Hua Zhu

**Affiliations:** 1 Department of Otorhinolaryngology, Head and Neck Surgery, The Second Affiliated Hospital, Jiangxi Medical College, Nanchang University, Nanchang, China; 2 Department of Anesthesiology, The First Hospital of Jinlin University, Changchun, China; 3 Department of Clinical Medicine, The First Clinical Medical College, Nanchang University, Nanchang, China; 4 China-Japan Union Hospital of Jilin University, Changchun, China

**Keywords:** airway allergic diseases, allergic rhinitis, composite study, dioctyl terephthalate, toxicological mechanism

## Abstract

**Background:**

Dioctyl terephthalate (DOTP), a common plasticizer alternative, has unclear immunotoxic effects on respiratory allergic diseases such as allergic rhinitis (AR), asthma (AS), chronic rhinosinusitis (CRS), and allergic bronchopulmonary aspergillosis (ABPA).

**Methods:**

We integrated network toxicology, target mining, bioinformatics, and machine learning to explore DOTP’s potential role in allergic airway diseases. Shared targets were analyzed via PPI networks, enrichment analyses, immune profiling, single-cell data, and molecular docking. Key genes were validated *in vitro* using human airway epithelial cells.

**Results:**

DOTP showed potential toxicity including carcinogenicity and nephrotoxicity. It shared 136 targets with allergic diseases, mainly involved in immune and apoptotic pathways. EGFR, BCL2, CFTR, SYK, and C3AR1 were identified as core genes. DOTP showed strong binding to these targets and induced cytotoxicity and gene expression changes in epithelial cells.

**Conclusion:**

DOTP may promote allergic airway diseases via immune-related and multi-target mechanisms. These findings highlight potential health risks of DOTP exposure and warrant further investigation.

## 1 Introduction

Dioctyl terephthalate (DOTP) is a new - type environmentally friendly plasticizer that has been widely used in multiple industrial fields in recent years (Harmon and Otter, 2022a; Harmon and Otter, 2022b). Compared with traditional phthalate plasticizers such as bis(2 - ethylhexyl) phthalate (DEHP), DOTP has advantages such as lower toxicity, less environmental residue, and higher safety, so it is widely recommended as a major substitute for DEHP (Rowdhwal and Chen, 2018). With its good thermal stability and plasticizing effect, DOTP has gradually become a mainstream product in fields such as food packaging, building materials, medical devices, and children’s toys (Rahman and Brazel, 2004).

At the same time, the potential pathogenic role of environmental pollutants in various respiratory allergic diseases has received increasing attention. These diseases include allergic rhinitis (AR), asthma (AS), chronic rhinosinusitis (CRS), and allergic bronchopulmonary aspergillosis (ABPA). Existing studies have shown that airborne particulate matter, organic compounds, and persistent pollutants can promote the occurrence and development of chronic respiratory inflammation by activating immune responses, disrupting epithelial barrier functions, or inducing oxidative stress (Mazzoli-Rocha et al., 2010; Bönisch et al., 2012; Kumar et al., 2014). Among them, phthalate plasticizers are considered one of the important potential risk factors due to their endocrine - disrupting and immunotoxic effects (Rowdhwal and Chen, 2018). Although DOTP exhibits low acute toxicity, research on its chronic toxic effects caused by long - term exposure, especially in the immune system, remains limited. Respiratory allergic diseases have a high degree of immune heterogeneity, and their pathogenesis involves abnormal infiltration of immune cells, disruption of the cytokine network, and abnormal activation of signal transduction pathways (Hirose et al., 2017; Scadding, 2014; Xie et al., 2024; Pawankar et al., 2015; Williams et al., 2012). Current toxicological research mainly focuses on its effects on reproduction and development (Liu et al., 2005; Xu et al., 2022), and there is a lack of systematic evaluation of whether it has chronic immunotoxicity similar to phthalate plasticizers such as DEHP.

Therefore, it is imperative to systematically evaluate the chronic toxicity and underlying mechanisms of DOTP exposure in relation to respiratory allergic diseases. To this end, our study integrates multi-omics computational approaches with experimental validation to elucidate these mechanisms, thereby providing a scientific foundation for the safety assessment and alternative strategies of DOTP in industrial applications.

Network toxicology, as an emerging toxicological method, integrates bioinformatics, systems biology, cheminformatics, genomics, proteomics, and other fields to construct relationships between compounds, toxicity, and targets (Souza et al., 2023). This interdisciplinary approach allows for systematic analysis of interactions between target proteins, helping to predict the molecular mechanisms of potential toxic substances in diseases. Machine learning provides efficient and objective analysis tools for high-dimensional gene feature selection and has been widely used in disease biomarker and environmental toxicity screening (Wang et al., 2021). Immune cell infiltration analysis offers a non-invasive means to study the immune microenvironment of diseases. The CIBERSORT algorithm can estimate the proportion distribution of 22 types of immune cells in tissue samples based on transcriptome data and has been widely used in immune-related diseases such as tumors and chronic inflammation (Chen B. et al., 2018). By comparing the differences in immune composition between disease and control groups, the dynamic changes of specific immune cells and their correlations with key genes under particular disease conditions can be revealed. Single-cell RNA sequencing technology (scRNA-seq) has played an important role in revealing tissue heterogeneity and cell subpopulation characteristics in recent years (Zhang et al., 2023; Van der Maaten and Hinton, 2008). By performing high-throughput expression analysis on individual cells and combining it with dimensionality reduction clustering (such as t-SNE) and cell annotation databases, the fine expression distribution of specific genes at the cellular level can be interpreted. The miRNA-transcription factor regulatory network (miRNA–TF network) provides important clues for revealing gene expression regulation mechanisms. miRNAs regulate mRNA stability and translation efficiency at the post-transcriptional level, while transcription factors regulate target gene expression at the transcription initiation stage. Constructing miRNA–mRNA, TF–mRNA, and TF–miRNA regulatory networks helps to systematically reveal the upstream regulatory patterns of key genes (Martinez and Walhout, 2009). Molecular docking technology is a core method in computer-aided drug design and is widely used to predict the binding modes and affinities between small molecules and proteins. By using tools such as AutoDock Vina for receptor preparation, grid setting, and docking scoring, potential binding sites and key interacting residues can be identified (Stanzione et al., 2021). Using these methods, researchers can gain a deeper understanding of how DOTP induces AR, AS, CRS, and ABPA by affecting specific molecular targets.

## 2 Materials and methods

### 2.1 Preliminary network analysis of DOTP toxicity

The chemical structure and standard SMILES format of DOTP were obtained by searching “dioctyl terephthalate” in the PubChem database (https://pubchem.ncbi.nlm.nih.gov/). Combining network search algorithms and biological toxicity prediction methods and integrating them into relevant software tools enabled us to predict the potential toxicity of DOTP based on its structural model. ProTox-3.0 (https://tox.charite.de/protox3/) and ADMETlab 3.0 (https://admetlab3.scbdd.com/server/screening) were used as preliminary screening tools to obtain basic information on DOTP-induced toxicity.

### 2.2 Identification of DOTP-related targets

Potential targets of DOTP were predicted using the Swiss Target Prediction (http://www.swisstargetprediction.ch/), STITCH (http://stitch.embl.de/) and ChEMBL (https://www.ebi.ac.uk/chembl/) databases, and the target names were standardized using the UniProt database (https://www.uniprot.org/). In Swiss Target Prediction and STITCH, the species was restricted to “*Homo sapiens*,” and in the ChEMBL database, “dioctyl terephthalate” was used as the keyword with the species restricted to “*H. sapiens*.”

### 2.3 Identification of disease-related targets

Using “allergic rhinitis,” “asthma,” “chronic rhinosinusitis,” and “allergic bronchopulmonary aspergillosis” as keywords, genes related to AR, AS, CRS, and ABPA were screened from the GeneCards (https://www.genecards.org/), TTD (https://db.idrblab.net/ttd/) and OMIM (https://www.omim.org) databases. Subsequently, using R (v4.4.3) and the “ggvenn” package, the Venn diagrams of DOTP-related targets and disease-related targets were drawn for AR, AS, CRS, and ABPA (Lin, 2026). The intersecting parts were considered as the potential targets of DOTP-induced AR, AS, CRS, and ABPA. Cytoscape (v3.10.3) was used for visualization to construct the network relationship between toxicant-target-disease.

### 2.4 Construction of protein-protein interaction (PPI) network and identification of potential key genes

The potential targets of DOTP-induced AR, AS, CRS, and ABPA were input into the STRING database (https://string-db.org/) to construct the interaction network of target genes. The minimum required interaction confidence score was set to 0.400 (medium confidence), and the “hide disconnected nodes” option was active. After exporting the data files, the “Analyze Network” tool in Cytoscape was used to analyze the topological structure of the constructed interaction network and calculate the “Degree” value of each node. The genes with the highest “Degree” values were selected as the potential key genes for each disease.

### 2.5 Functional and pathway enrichment analysis of potential targets

Using R (v4.4.3) and the “clusterProfiler” and “OrgDb” packages, we performed gene ontology (GO) and Kyoto Encyclopedia of Genes and Genomes (KEGG) enrichment analyses on the intersecting targets to elucidate the key signaling pathways and identify biological processes (BP, referring to dynamic biological events involved in gene function), cellular components (CC, denoting the subcellular structure or location where gene-encoded proteins act), and molecular functions (MF, representing the biochemical activity of proteins encoded by genes) related to the potential therapeutic targets of AR, AS, CRS, and ABPA (Yu et al., 2012).

### 2.6 Machine learning

For subsequent machine learning studies, we obtained the core gene expression matrices from the following GEO datasets: AR (GSE206149), AS (GSE51392), CRS (GSE136825), and ABPA (GSE78000). These datasets provided the transcriptomic profiles necessary for our analysis. To further identify core genes with therapeutic potential in the PPI network, we constructed four classical machine learning models using the 12 potential key genes as candidate features, including LASSO regression (Least Absolute Shrinkage and Selection Operator, https://glmnet.stanford.edu/), Random Forest (RF, https://cran.r-project.org/web/packages/randomForest/index.html), Support Vector Machine (SVM, https://www.csie.ntu.edu.tw/∼cjlin/libsvm/)and Extreme Gradient Boosting (XGBoost, https://xgboost.ai/). LASSO regression selected the most representative feature genes through 10-fold cross-validation to determine the optimal penalty parameter λ. RF and XGBoost models assessed the importance of each feature based on its contribution to classification performance and ranked them accordingly. SVM used recursive feature elimination (RFE) combined with cross-validation strategies to determine the optimal feature subset. Each model output the feature importance rankings, and key genes that stood out in multiple algorithms were identified by cross-comparing the results of the four models.

### 2.7 Immune cell infiltration analysis

To evaluate the relationship between key genes and the immune microenvironment, we used the CIBERSORT algorithm to estimate the composition of immune cells in the samples (Newman et al., 2015). Comparisons were made between AR, AS, CRS, and ABPA and normal controls (NC), with immune cell proportion bar charts and grouped difference box plots drawn to assess the expression differences of various immune cells among the groups. Significant differences were assessed using the Wilcoxon rank-sum test, with a significance level set at P < 0.05.

### 2.8 Single-cell RNA sequencing data analysis

To further investigate the expression distribution of key genes in different cell types, we downloaded allergic airway epithelial cell scRNA-seq data (45,100 cells and 27,517 genes) from the Single Cell Portal platform (http://singlecell.broadinstitute.org). Additional quality control criteria included: number of detected genes per cell (200–6,000), UMI count per cell (500–30,000), and ribosomal gene percentage <60% to exclude doublets and low-quality cells. The dataset included samples from 3 healthy controls and 5 patients with allergic airway diseases (2 AR, 1 AS, 1 CRS, 1 ABPA), with epithelial cells isolated from nasal and bronchial mucosal tissues via bronchoscopy and nasal scraping. The scRNA-seq data were integrated using R and the Seurat package. Cells with mitochondrial gene percentage exceeding 10% were excluded using the “PercentageFeatureSet function”. Validated by large-sample data analysis, this threshold is suitable for quality control of single-cell sequencing of human airway epithelial cells. It can not only effectively filter out low-quality cells caused by cell damage or apoptosis, but also avoid the erroneous deletion of metabolically active normal epithelial cells, ensuring the accuracy and integrity of the data (Osorio and Cai, 2021). The data were normalized using the “LogNormalize” method (Butler et al., 2018), High variability genes were selected using the FindVariableFeatures function, followed by principal component analysis (PCA) and non-linear dimensionality reduction clustering (t-SNE). To identify standard markers for distinguishing different cell types, we referred to the cell marker database (http://xteam.xbio.top/CellMarker/index.jsp#). Subsequently, we visualized the expression differences of the key genes identified by machine learning in various cell types. Statistical analysis of subgroup differential expression was conducted via the Wilcoxon rank-sum test, with significance defined as adjusted *P* < 0.05 and |log_2_FC| > 0.5. The results demonstrated that EGFR was significantly upregulated in olfactory epithelial basal cells with log_2_FC = 1.23 and adjusted *P* = 2.1 × 10^−8^; BCL2 was moderately expressed in T Cells with log_2_FC = 0.67 and adjusted *P* = 3.5 × 10^−4^ as well as in plasma cells with log_2_FC = 0.58 and adjusted *P* = 1.8 × 10^−3^; CFTR was highly enriched in goblet cells with log_2_FC = 1.56 and adjusted *P* = 7.3 × 10^−12^ and ciliated cells with log_2_FC = 1.19 and adjusted *P* = 4.9 × 10^−9^; SYK was significantly overexpressed in myeloid cells with log_2_FC = 1.03 and adjusted *P* = 5.2 × 10^−7^ and mast cells with log_2_FC = 0.91 and adjusted *P* = 8.6 × 10^−6^; C3AR1 was specifically upregulated in neutrophils with log_2_FC = 1.37 and adjusted *P* = 3.8 × 10^−10^ and monocytes with log_2_FC = 1.05 and adjusted *P* = 6.1 × 10^−8^.

### 2.9 miRNA-TFs

To further reveal the regulatory mechanisms of key genes at the transcriptional and post-transcriptional levels, we utilized databases to predict upstream regulatory miRNAs related to key genes (EGFR, BCL2, CFTR, SYK, C3AR1), incorporating only miRNAs predicted by multiple databases to enhance prediction accuracy. Subsequently, transcription factors (TFs) associated with these key genes and miRNAs were extracted from relevant databases. The miRNA–mRNA, TF–mRNA, and TF–miRNA regulatory relationships were imported into Cytoscape (version 3.10.3) for network construction and visualization, with node types and regulatory directions annotated to display the upstream regulatory patterns of key genes.

### 2.10 GSEA

To explore the potential biological functions of key genes in regulatory networks, we performed Gene Set Enrichment Analysis (GSEA) on the key genes. We obtained the GSEA software (version 4.4.0) from the GSEA website (http://software.broadinstitute.org/gsea/index.jsp) (Subramanian et al., 2005; Mootha et al., 2003) and downloaded the c2. cp.kegg.v7.4. symbols.gmt subset from the Molecular Signatures Database (http://www.gsea-msigdb.org/gsea/downloads.jsp) (Liberzon et al., 2011) to evaluate relevant pathways and molecular mechanisms. Based on gene expression profiles and phenotypic grouping, we set the minimum gene set size to 5 and the maximum to 5000, with 1,000 permutations. P-values less than 0.05 and FDR less than 0.3 were considered statistically significant.

### 2.11 Molecular docking

Molecular docking methods were employed to analyze the molecular interactions between DOTP and key gene proteins by predicting binding modes and affinities. The crystal structures of core proteins were downloaded from the RCSB PDB database (https://www.rcsb.org/). Water molecules and original ligands in the target proteins were removed using PyMOL, followed by hydrogenation, charge calculation, and non-polar hydrogen combination using AutoDock Tools (https://autodock.scripps.edu/). After determining the grid box size and genetic algorithm, molecular docking was performed using AutoDock Vina (Trott and Olson, 2010).

### 2.12 *In vitro* experiments

#### 2.12.1 Cell strains and culture conditions

Human nasal mucosal epithelial cell line HNEpC and lung epithelial cell line BEAS2B were purchased from the PromoCell GmbH (Heidelberg, Germany) and confirmed to be contamination-free by STR identification (Shanghai Yihe Applied Biotechnology Co., Ltd., Shanghai, China).

For the initial cytotoxicity screening, a range of DOTP concentrations (0, 50, 100, and 200 μmol/L) were prepared. Based on the CCK-8 assay results (Section 3.10), 100 μmol/L was selected as the standard concentration for subsequent functional experiments (cell morphology observation and qPCR analysis), with a uniform treatment duration of 48 h. The culture conditions were 37 °C, 5% CO_2_ in an incubator, using 1,640 culture medium (Gibco, Thermo Fisher Scientific, United States) supplemented with 5% fetal bovine serum (FBS; Gibco, Thermo Fisher Scientific, United States) and 1% penicillin–streptomycin solution (antibiotics; Gibco, Thermo Fisher Scientific, United States).

#### 2.12.2 CCK8 assay for cell proliferation viability

Healthy HNEpC and BEAS2 B cells were digested with trypsin-EDTA solution (Gibco, Thermo Fisher Scientific, United States) for 3–5 min. The cell suspension was centrifuged at 1,000 rpm for 5 min, the supernatant was discarded, and the cells were resuspended in an appropriate amount of culture medium. A 10 μL cell suspension was counted using a cell counter, and cells were seeded at a density of 1 × 10^4^ cells per well in a 96-well plate. After cell attachment, different concentrations of DOTP were added for 24 h, with 3-5 replicates per group to ensure stable results. The growth of cells in each well was recorded under an optical microscope, followed by the addition of 10 µL of CCK-8 solution (Dojindo Laboratories, Kumamoto, Japan) and incubation at 37 °C for 1–4 h. All operations were strictly conducted under sterile conditions. Finally, the absorbance was measured at 450 nm using a microplate reader.

#### 2.12.3 Microscopic observation of cell state

Cells treated with DOTP at 100 μmol/L for 48 h were observed under an optical microscope for cell state and quantity at magnifications of ×100 and ×400.

#### 2.12.4 PCR detection of cellular mRNA

After 48 h of DOTP treatment, total RNA was extracted from cultured HNEpC and BEAS2 B cells using TRIzol® reagent (Invitrogen, Thermo Fisher Scientific, United States), and the concentration and purity (A260/A280 = 1.8–2.0) were determined using NanoDrop 2000spectrophotometer (Thermo Fisher Scientific, United States). One microgram of RNA was reverse-transcribed into cDNA using the PrimeScript™ RT reagent kit (Takara). Specific primers for target genes and the internal reference gene GAPDH (Table 1) were designed and used for qPCR with SYBR Green on the QuantStudio 5 system (Applied Biosystems). The reaction conditions were as follows: 95 °C for 5 min, followed by 40 cycles of 95 °C for 10 s, 60 °C for 30 s, and 72 °C for 30 s. The relative expression levels of the genes were calculated using the ΔΔCt method. The experiments were repeated three times, and the data are presented as mean ± standard deviation. Intergroup differences were analyzed using Student’s t-test (P < 0.05 was considered significant).

**TABLE 1:** The following primers were used for RT-qPCR.

Gene name	Primer direction	Primer sequence (5'→3′)
EGFR	Forward	CAG​CGC​TAC​CTT​GTC​ATT​CAG
	Reverse	GGC​AGT​CAC​AGA​CCA​AAG​GT
BCL-2	Forward	GGT​GGA​CAA​CAT​CGC​TCT​GT
	Reverse	CAG​CCA​GGA​GAA​ATC​AAA​CAG
CFTR	Forward	TGC​CAC​CTA​TCA​GAG​CCA​AC
	Reverse	TGT​GGT​AAG​CCA​TGC​TGT​TC
SYK	Forward	TGG​ACC​TGA​AGG​ACA​AGC​TG
	Reverse	TCC​AGG​TCC​ACG​TAG​TTG​CT
C3AR1	Forward	TCT​GGG​CTT​CCT​CAT​CCT​CT
	Reverse	AGG​CAG​GTG​ATG​TTG​AGG​TG
GAPDH	Forward	GGA​GCG​AGA​TCC​CTC​CAA​AAT
	Reverse	GGC​TGT​TGT​CAT​ACT​TCT​CAT​GG

#### 2.12.5 Data analysis

Data are presented as mean ± standard deviation (SD). All analyses were performed using GraphPad Prism version 6.0. Independent sample t-tests were used for comparisons between two groups, and one-way ANOVA was used for comparisons among multiple groups. Post-hoc tests were conducted using Tukey’s HSD test (for homogeneity of variance) or Dunnett’s T3 test (for heterogeneity of variance).

## 3 Results

### 3.1 DOTP toxicity assessment

After integrating the output from ProTox-3.0 (https://tox.charite.de/protox3/) and ADMETlab 3.0 (https://admetlab3.scbdd.com/server/screening), we obtained a toxicity profile for DOTP. The toxicity model indicated associations with carcinogenicity, blood-brain barrier permeability, nephrotoxicity, hERG blockade, skin sensitization, and eye irritation.

### 3.2 Identification of potential targets for DOTP-Induced AR, AS, CRS, and ABPA

**FIGURE 1:**
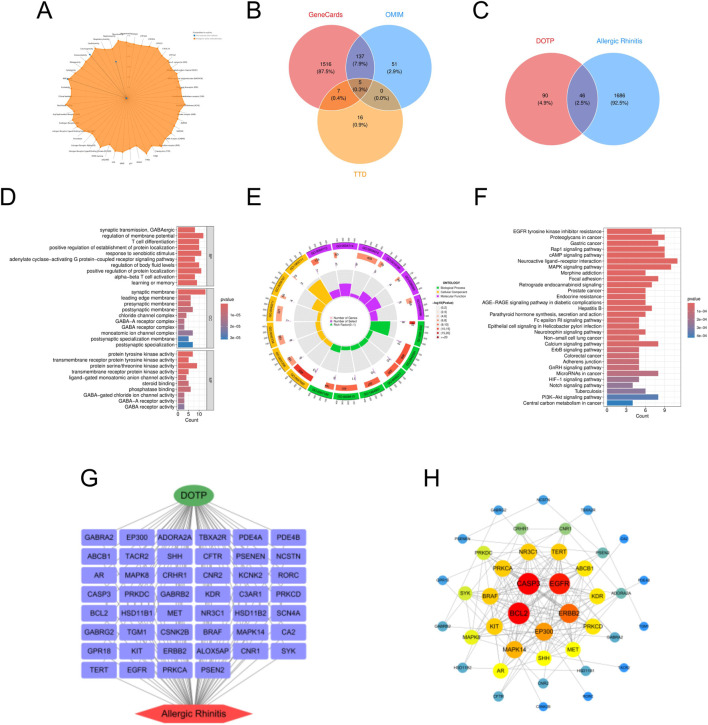
Network toxicology analysis of AR due to DOTP. **(A)** Toxicological activation targets of DOTP. **(B)** Pathogenic targets of AR. **(C)** Venn diagram of the intersection of AR pathogenic targets and DOTP toxicological activation targets. **(D,E)** GO enrichment analysis of intersecting genes. **(F)** KEGG enrichment analysis of intersecting genes. **(G,H)** Protein-protein interaction network analysis of intersecting genes.

**FIGURE 2:**
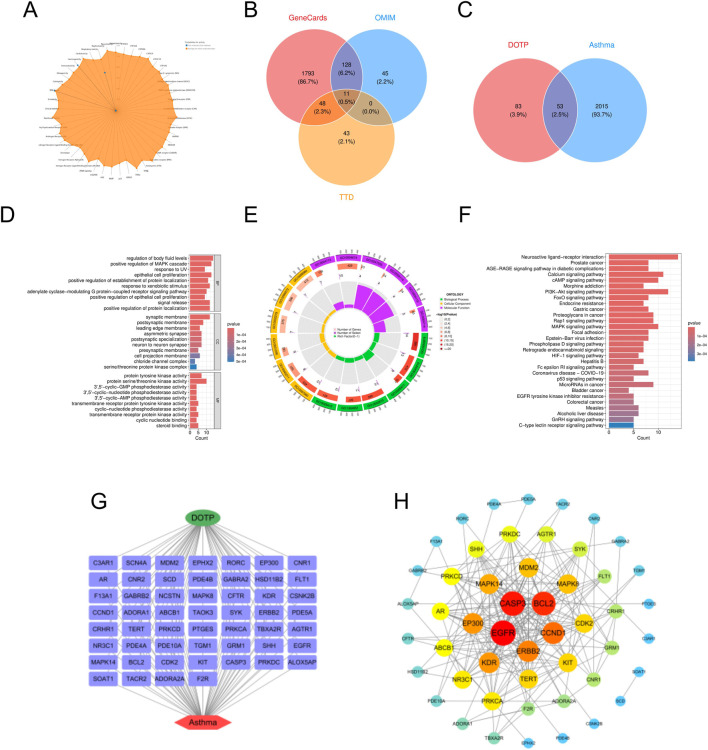
Network toxicology analysis of AS due to DOTP. **(A)** Toxicological activation targets of DOTP. **(B)** Pathogenic targets of AS. **(C)** Venn diagram of the intersection of pathogenic targets of AS and toxicologically activated targets of DOTP. **(D,E)** GO enrichment analysis of intersecting genes. **(F)** KEGG enrichment analysis of intersecting genes. **(G,H)** Protein-protein interaction network analysis of intersecting genes.

**FIGURE 3:**
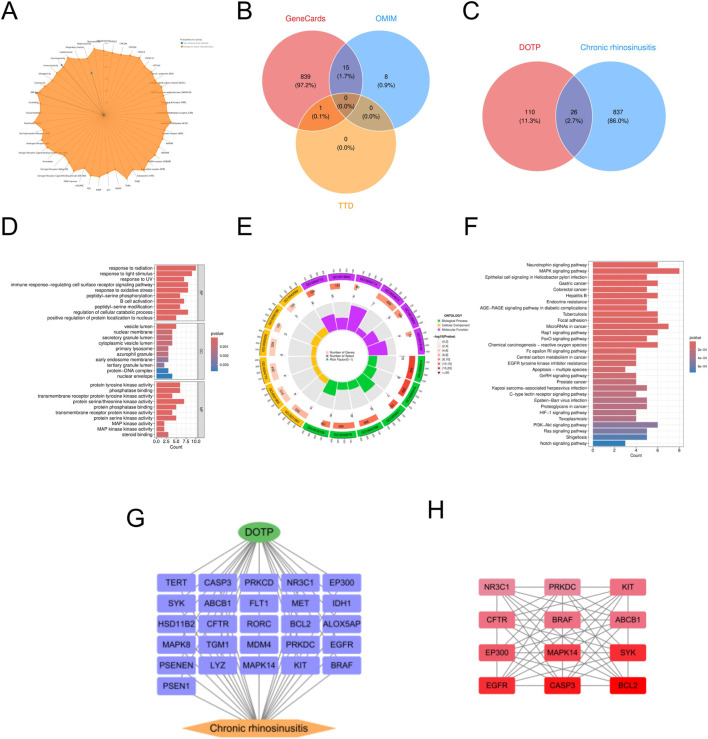
Network toxicology analysis of CRS due to DOTP. **(A)** Toxicological activation targets of DOTP. **(B)** Pathogenic targets of CRS. **(C)** Venn diagram of the intersection of CRS pathogenic targets and DOTP toxicological activation targets. **(D,E)** GO enrichment analysis of intersecting genes. **(F)** KEGG enrichment analysis of intersecting genes. **(G,H)** Protein-protein interaction network analysis of intersecting genes.

**FIGURE 4:**
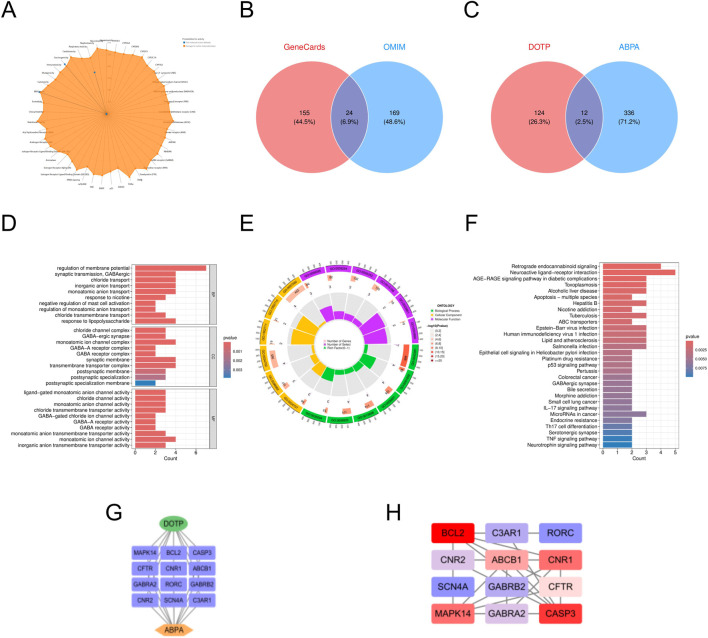
Network toxicology analysis of ABPA due to DOTP. **(A)** Toxicological activation targets of DOTP. **(B)** Pathogenic targets of ABPA. **(C)** Venn diagram of the intersection of ABPA pathogenic targets and DOTP toxicological activation targets. **(D,E)** GO enrichment analysis of intersecting genes. **(F)** KEGG enrichment analysis of intersecting genes. **(G,H)** Protein-protein interaction network analysis of intersecting genes.

In this study, we retrieved the standard structure and specific molecular information of DOTP from the PubChem database. We identified 136 potential targets for DOTP using the ChEMBL, STITCH, and SwissTargetPrediction databases. From the GeneCards, OMIM, and TTD databases, we obtained 1732 potential targets for AR, 2068 for AS, 863 for CRS, and 348 for ABPA. By integrating these target sets, we identified 46 intersecting targets for DOTP and AR, 53 for DOTP and AS, 26 for DOTP and CRS, and 12 for DOTP and ABPA, which were considered as potential targets for DOTP-induced AR (Figure 1), AS (Figure 2), CRS (Figure 3), and ABPA (Figure 4).

### 3.3 Construction of PPI network and identification of potential key genes

We constructed four protein-protein interaction (PPI) networks using the STRING database and analyzed the topological properties of the network nodes, including degree, betweenness centrality, and closeness centrality, using Cytoscape (v3.10.3). The top twelve genes with the highest degree values were selected to generate the optimized PPI network diagrams, which specifically displayed the interactions among these potential key genes (Tables 2–5). The potential key genes for AR (Figure 1) were BCL2, CASP3, EGFR, ERBB2, EP300, MAPK14, KIT, BRAF, PRKCA, NR3C1, TERT, and ABCB1. For AS (Figure 2), the potential key genes were EGFR, CASP3, BCL2, CCND1, ERBB2, KDR, EP300, MAPK14, MDM2, MAPK8, CDK2, and KIT. For CRS (Figure 3), the potential key genes were BCL2, CASP3, EGFR, SYK, MAPK14, EP300, ABCB1, BRAF, CFTR, KIT, PRKDC, and NR3C1. For ABPA (Figure 4), the potential key genes were BCL2, CASP3, MAPK14, CNR1, ABCB1, CFTR, CNR2, GABRA2, GABRB2, C3AR1, RORC, and SCN4A.

**TABLE 2:** Protein-protein interaction analysis of the top 10 key genes screened for DOTP-AR.

Name	Degree	Betweenness centrality	Closeness centrality
BCL2	21	0.160560504	0.615384615
CASP3	21	0.130681402	0.606060606
EGFR	20	0.094982379	0.579710145
ERBB2	17	0.015951872	0.506329114
EP300	15	0.110477902	0.533333333
MAPK14	14	0.134068848	0.540540541
TERT	13	0.006287139	0.470588235
BRAF	13	0.010842676	0.519480519
PRKCA	13	0.101573057	0.487804878
KIT	13	0.013962658	0.49382716

**TABLE 3:** Protein-protein interaction analysis of the top 10 key genes screened for DOTP-AS.

Name	Degree	Betweenness centrality	Closeness centrality
EGFR	24	0.08911722849049397	0.5662650602409639
CASP3	23	0.12839795624254458	0.5802469135802469
BCL2	22	0.04669411301002421	0.5529411764705883
CCND1	19	0.03880135564918172	0.5280898876404494
ERBB2	18	0.013147418929102556	0.49473684210526314
EP300	17	0.06454804189540544	0.49473684210526314
KDR	17	0.1775400581020378	0.5164835164835164
MAPK14	16	0.09468669114830078	0.5164835164835164
MAPK8	15	0.025951338208507493	0.48958333333333337
MDM2	15	0.007839359735751965	0.48958333333333337

**TABLE 4:** Protein-protein interaction analysis of the top 10 key genes screened for DOTP-CRS.

Name	Degree	Betweenness centrality	Closeness centrality
BCL2	24	0.080582505	0.961538462
CASP3	23	0.082467731	0.925925926
SYK	22	0.042463458	0.892857143
EGFR	22	0.065544654	0.892857143
MAPK14	21	0.024963458	0.862068966
EP300	20	0.020609367	0.833333333
BRAF	19	0.009757275	0.806451613
KIT	19	0.02285119	0.806451613
CFTR	19	0.00868741	0.806451613
ABCB1	19	0.00868741	0.806451613

**TABLE 5:** Protein-protein interaction analysis of the top 10 key genes screened for DOTP-ABPA.

Name	Degree	Betweenness centrality	Closeness centrality
BCL2	8	0.3	0.785714286
CASP3	7	0.118181818	0.733333333
CNR1	6	0.225757576	0.6875
MAPK14	6	0.196969697	0.6875
ABCB1	5	0.077272727	0.647058824
CFTR	4	0	0.578947368
CNR2	3	0	0.55
GABRA2	3	0.027272727	0.5
GABRB2	2	0	0.458333333
C3AR1	2	0	0.47826087

### 3.4 Functional and pathway enrichment analysis of potential targets

In the Gene Ontology (GO) analysis, we sorted GO terms based on FDR values and selected the top 10 terms with the lowest FDR values in BP, CC, and MF for visualization in the enrichment analysis diagrams (Figures 1–4).

For AR (Figure 1), we performed GO analysis on the 46 intersecting targets. In the biological process (BP) category, “synaptic transmission, GABAergic,” “T Cell differentiation,” and “response to xenobiotic stimulus” were significantly enriched. This suggests that DOTP may mediate the occurrence and development of AR by modulating specific neural signaling pathways, such as GABAergic synaptic transmission identified in our GO analysis, and immune cell differentiation. In the cellular component (CC) category, “synaptic membrane,” “postsynaptic membrane,” and “monoatomic ion channel complex” were significantly enriched, indicating that the targets may play important roles in the synaptic structure and ion channel functions within the nervous system. In the molecular function (MF) category, “protein tyrosine kinase activity,” “protein serine/threonine kinase activity,” and “transmembrane receptor protein tyrosine kinase activity” were significantly enriched, suggesting that DOTP may influence cell signaling and immune responses by regulating protein phosphorylation. Subsequent KEGG pathway enrichment analysis revealed that the significantly enriched pathways were mainly concentrated in immune regulation, neural modulation, and tumor-related signaling pathways. The most significant pathways included “MAPK signaling pathway,” “Neuroactive ligand-receptor interaction,” and “Fc epsilon RI signaling pathway.” These pathways are closely related to allergic reactions in the immune system, especially the FcεRI signaling pathway, which is involved in IgE-mediated allergic reactions. Additionally, “Adherens junction” and “Endocrine resistance” were enriched, indicating that DOTP may influence the pathological process of AR by affecting cell adhesion and hormone response.

For AS (Figure 2), we performed GO analysis on the 53 intersecting targets. In the biological process (BP) category, “regulation of body fluid levels,” “positive regulation of MAPK cascade,” “positive regulation of epithelial cell proliferation,” and “response to xenobiotic stimulus” were significantly enriched. These enrichment terms suggest that DOTP may participate in the immune-inflammatory response of AS by regulating body fluid homeostasis, cell proliferation, and cellular stress responses. In the cellular component (CC) category, “synaptic membrane,” “postsynaptic membrane,” and “asymmetric synapse” were significantly enriched, which are closely related to neural signal transmission and synaptic transmission, indicating that neuroimmune responses may be involved in AS. In the molecular function (MF) category, “protein tyrosine kinase activity,” “protein serine/threonine kinase activity,” and “transmembrane receptor protein tyrosine kinase activity” were significantly enriched, suggesting that these targets may participate in inflammatory responses and immune responses by regulating cell signaling. Subsequent KEGG pathway enrichment analysis revealed that the significantly enriched pathways were mainly concentrated in immune responses, cell proliferation, signal transduction, and chronic disease-related pathways. “Neuroactive ligand-receptor interaction,” “AGE–RAGE signaling pathway in diabetic complications,” “cAMP signaling pathway,” “MAPK signaling pathway,” and “FoxO signaling pathway” were significantly enriched, indicating that DOTP may regulate the pathological process of AS through neural modulation, signal transduction, and cell proliferation pathways.

For CRS (Figure 3), we performed GO analysis on the 26 intersecting targets. In the biological process (BP) category, “immune response–regulating cell surface receptor signaling pathway,” “response to oxidative stress,” “response to UV,” and “peptidyl–serine phosphorylation” were significantly enriched, suggesting that DOTP may participate in the pathological mechanism of CRS by regulating immune responses, oxidative stress responses, and protein phosphorylation processes. In the cellular component (CC) category, “vesicle lumen,” “nuclear membrane,” and “primary lysosome” were significantly enriched, suggesting that DOTP may influence the transport and metabolism of substances within cells by affecting the functions of intracellular vesicles and lysosomes. In the molecular function (MF) category, “protein tyrosine kinase activity,” “protein serine/threonine kinase activity,” and “phosphatase binding” were significantly enriched, suggesting that DOTP may mediate cell signaling and immune responses by regulating protein phosphorylation and dephosphorylation processes. Subsequent KEGG pathway enrichment analysis revealed that the significantly enriched pathways were mainly concentrated in immune regulation, oxidative stress, cell apoptosis, and tumor-related signaling pathways. “Neurotrophin signaling pathway,” “MAPK signaling pathway,” “PI3K-Akt signaling pathway,” and “Fc epsilon RI signaling pathway” were significantly enriched, indicating that these pathways are closely related to immune responses, cell survival, and proliferation, suggesting that DOTP may play a role in the immunopathological process of CRS by regulating these signaling pathways.

For ABPA (Figure 4), we performed GO analysis on the 12 intersecting targets. In the biological process (BP) category, “regulation of membrane potential,” “synaptic transmission, GABAergic,” “chloride transport,” and “response to nicotine” were significantly enriched, suggesting that DOTP may participate in the pathogenesis of ABPA by regulating ion channel activity and neurotransmitter-related pathways, possibly involving neuro-immune interaction regulatory mechanisms. In the cellular component (CC) category, “GABA–ergic synapse,” “synaptic membrane,” and “postsynaptic specialization membrane” were significantly enriched. This indicates that DOTP may play a role in the interaction between the central nervous system and the immune system in ABPA. In the molecular function (MF) category, “ligand-gated monoatomic anion channel activity,” “GABA–A receptor activity,” and “chloride channel activity” were significantly enriched, suggesting that DOTP may influence the airway hyperresponsiveness and inflammatory response in ABPA by regulating ion channels and neurotransmitter receptors. Subsequent KEGG pathway enrichment analysis revealed that the significantly enriched pathways were mainly concentrated in inflammation, infection, neurotransmitter signaling, and metabolism-related pathways. “Retrograde endocannabinoid signaling,” “Neuroactive ligand-receptor interaction,” “AGE-RAGE signaling pathway in diabetic complications,” and “Apoptosis - multiple species” were significantly enriched. These pathways involve inflammatory responses, cell death, and neural regulation, suggesting that DOTP may influence the immune and tissue damage processes in ABPA through these mechanisms. Additionally, enrichment was observed in “Nicotine addiction,” “GABAergic synapse,” “IL-17 signaling pathway,” and “TNF signaling pathway,” among other inflammation and neuroimmune-related pathways, further emphasizing that DOTP may play a potential regulatory role in the pathogenesis of ABPA through mechanisms involving immune-inflammatory responses, neurotransmitter pathways, and oxidative stress.

### 3.5 Machine learning

To further identify core targets closely related to the pathogenesis of AR from the 10 potential key genes, we applied four commonly used machine learning algorithms (LASSO, SVM, Random Forest, XGBoost) for feature selection and importance assessment. The criteria for selecting key genes were set as MeanDecreaseGini >1.5 for LASSO regression, Weight Importance ≥0.15 for SVM, Weight Importance ≥0.1 for XGBoost, and RF > 0.1 with an AUC value >0.7 as the final selection criteria.

**FIGURE 5:**
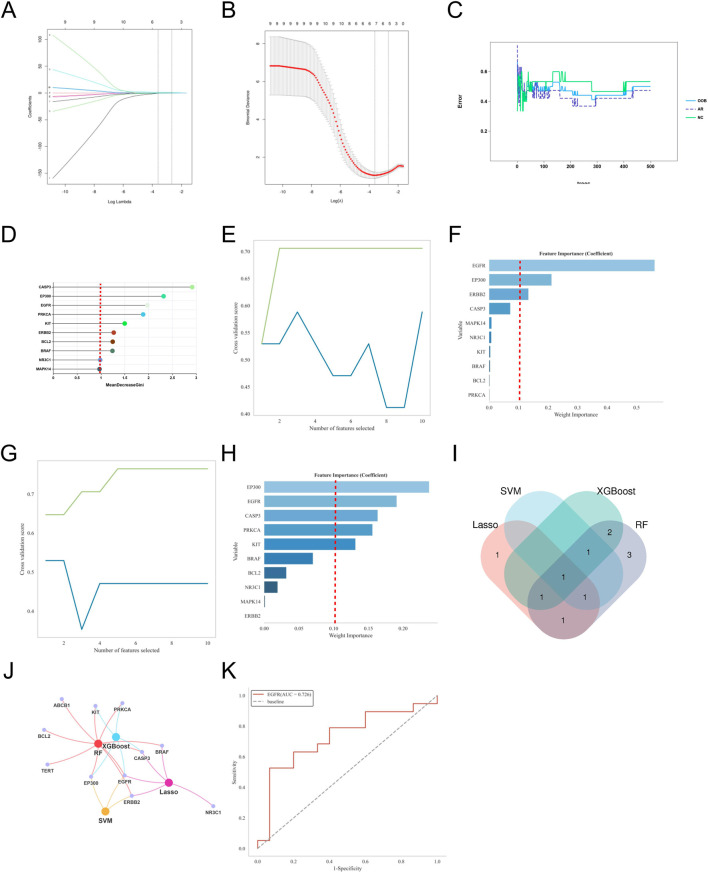
Machine learning screening of key genes for DOTP-AR. **(A,B)** Lasso regression analysis. **(C,D)** Random forest (RF) analysis. **(E,F)** Support vector machine (SVM) analysis. **(G,H)** EXtreme Gradient Boosting (XGBoot) analysis. **(I)** Venn diagrams of key genes obtained by four machine learning methods. **(J)** Network Venn diagram of key genes obtained by four machine learning methods. **(K)** ROC curves of key genes.

**FIGURE 6:**
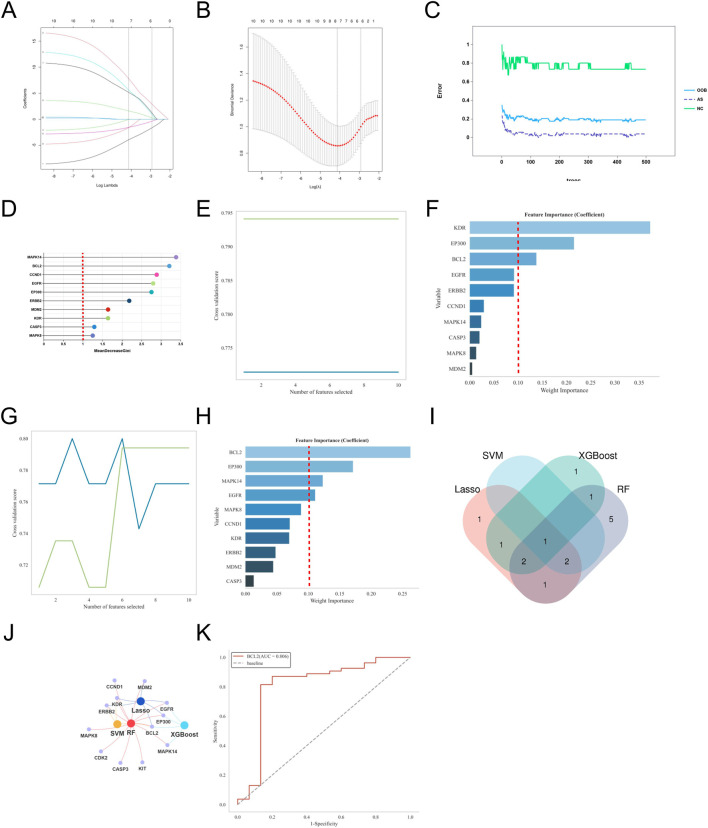
Machine learning screening of key genes for DOTP-AS. **(A,B)** Lasso regression analysis. **(C,D)** Random forest (RF) analysis. **(E,F)** Support vector machine (SVM) analysis. **(G,H)** EXtreme Gradient Boosting (XGBoot) analysis. **(I)** Venn diagrams of key genes obtained by four machine learning methods. **(J)** Network Venn diagram of key genes obtained by four machine learning methods. **(K)** ROC curves of key genes.

**FIGURE 7:**
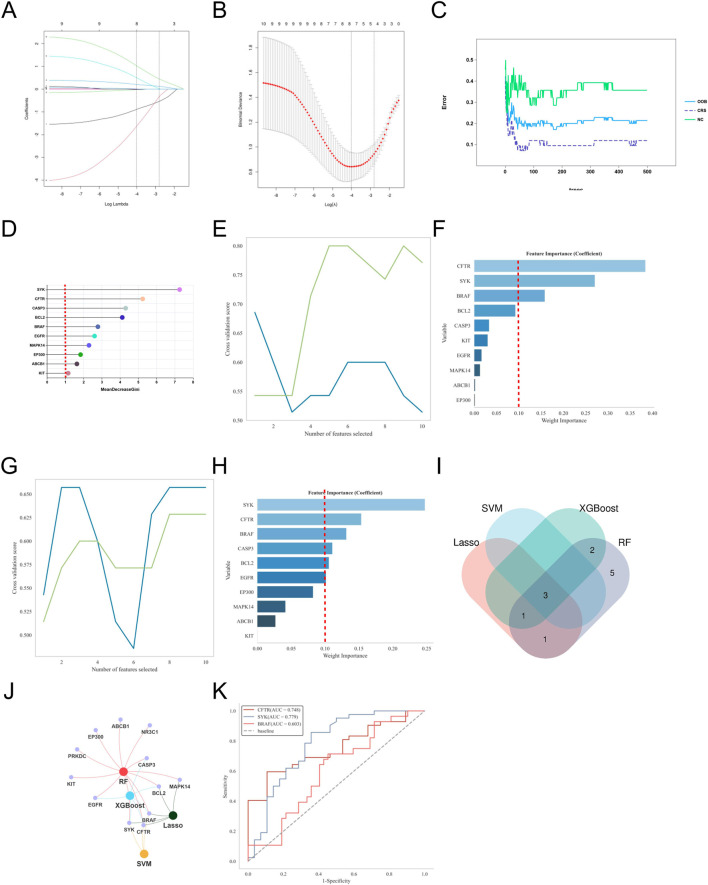
Machine learning screening of key genes for DOTP-CRS. **(A,B)** Lasso regression analysis. **(C,D)** Random forest (RF) analysis. **(E,F)** Support vector machine (SVM) analysis. **(G,H)** EXtreme Gradient Boosting (XGBoot) analysis. **(I)** Venn diagrams of key genes obtained by four machine learning methods. **(J)** Network Venn diagram of key genes obtained by four machine learning methods. **(K)** ROC curves of key genes.

**FIGURE 8:**
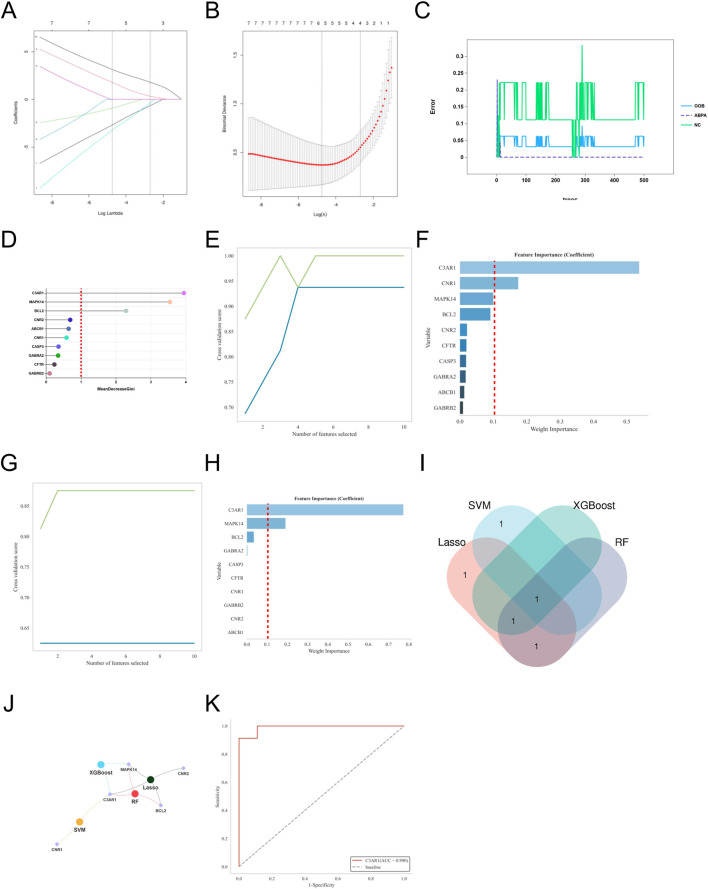
Machine learning screening of key genes for DOTP-ABPA. **(A,B)** Lasso regression analysis. **(C,D)** Random forest (RF) analysis. **(E,F)** Support vector machine (SVM) analysis. **(G,H)** EXtreme Gradient Boosting (XGBoot) analysis. **(I)** Venn diagrams of key genes obtained by four machine learning methods. **(J)** Network Venn diagram of key genes obtained by four machine learning methods. **(K)** ROC curves of key genes.

Based on the selection criteria, for AR, LASSO regression identified 5 key genes, SVM identified 3 key genes, XGBoost identified 5 key genes, and Random Forest identified 10 key genes (Figure 5). Through Venn diagram analysis, the key gene for DOTP-induced AR was identified as EGFR. For AS, LASSO regression identified 6 key genes, SVM identified 3 key genes, XGBoost identified 4 key genes, and Random Forest identified 10 key genes (Figure 6). Through Venn diagram analysis, the key gene for DOTP-induced AS was identified as BCL2. For CRS, LASSO regression identified 5 key genes, SVM identified 3 key genes, XGBoost identified 6 key genes, and Random Forest identified 10 key genes (Figure 7). Through Venn diagram analysis, the key genes for DOTP-induced CRS were identified as CFTR, SYK, and BRAF. For ABPA, LASSO regression identified 4 key genes, SVM identified 2 key genes, XGBoost identified 2 key genes, and Random Forest identified 3 key genes. Through Venn diagram analysis, the key gene for DOTP-induced ABPA was identified as C3AR1 (Figure 8).

Ultimately, the set of key genes identified for DOTP-induced AR, AS, CRS, and ABPA were EGFR (AR), BCL2 (AS), CFTR (CRS), SYK (CRS), and C3AR1 (ABPA).

### 3.6 Immune cell infiltration analysis and single-cell transcriptome expression analysis

**FIGURE 9:**
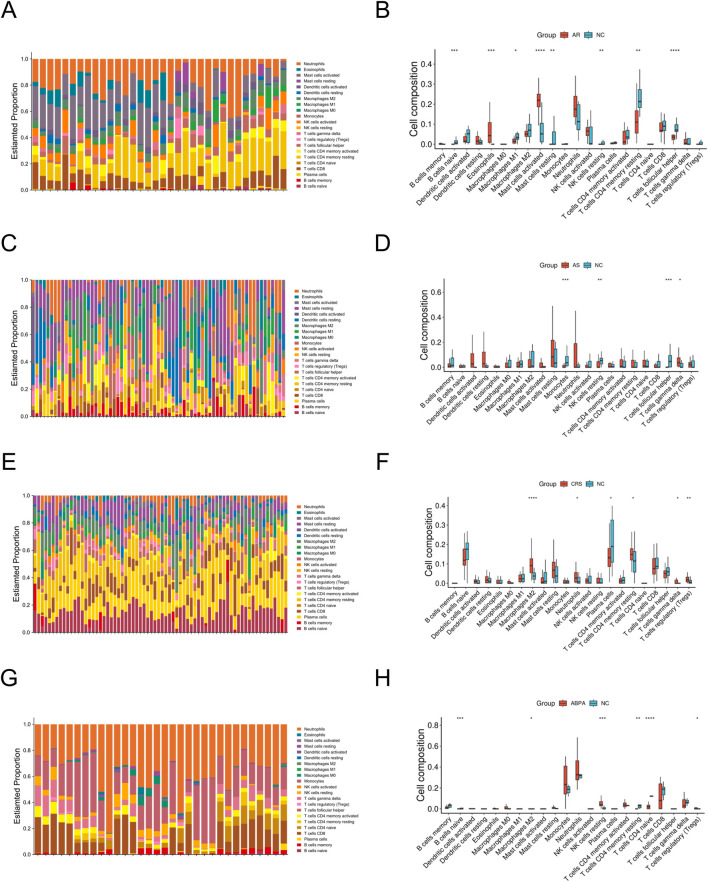
Immune infiltration analysis. **(A,B)** Immunoinfiltration analysis of AR. **(C,D)** Immune infiltration analysis of AS. **(E,F)** Immune infiltration analysis of CRS. **(G,H)** Immune infiltration analysis of ABPA.

We utilized the CIBERSORT algorithm to analyze the immune infiltration in samples from patients with AR, AS, CRS, and ABPA compared to normal controls (Figure 9). In the AR group, activated dendritic cells, monocytes, M0 and M1 macrophages, and neutrophils were significantly upregulated, while the proportions of memory B cells and CD8^+^ T Cells were markedly decreased, indicating that AR may be associated with innate immune activation and adaptive immune suppression. The AS group exhibited significant increases in neutrophils, M0 macrophages, and regulatory T Cells (Tregs), suggesting the presence of an immunosuppressive microenvironment. The CRS group showed higher proportions of M1 macrophages and neutrophils, with differential distribution of adaptive immune cells, reflecting the complex regulation of the immune system in the context of chronic inflammation. The ABPA group was characterized by a significant increase in neutrophils, along with an increase in Tregs and activated NK cells, indicating a dual feature of allergic and immune-activating characteristics.

**FIGURE 10:**
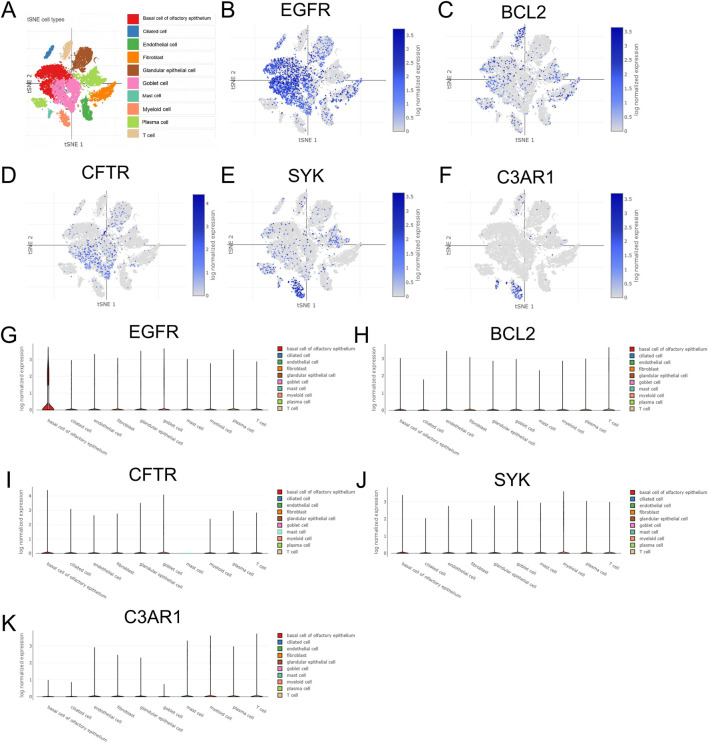
Single-cell sequencing analysis of key genes in allergic airway epithelia. **(A)** Overall cellular distribution. **(B,G)** Cellular distribution of EGFR genes. **(C,H)** Cellular distribution of BCL2 gene. **(D,I)** Cellular distribution of the CFTR gene. **(E,J)** Cellular distribution of the SYK gene. **(F,K)** Cellular distribution of the C3AR1 gene.

Based on these findings, we further conducted single-cell transcriptome expression analysis to clarify the expression distribution of key genes in specific cell types (Figure 10). Through t-SNE dimensionality reduction clustering, 11 major cell clusters were identified, including olfactory epithelial basal cells, ciliated cells, goblet cells, fibroblasts, plasma cells, T Cells, myeloid cells, mast cells, etc. The analysis revealed that EGFR was highly expressed in olfactory epithelial basal cells, suggesting its potential involvement in mucosal epithelial repair and structural remodeling, particularly in AR and CRS. BCL2 expression was relatively low, mainly concentrated in T Cells and plasma cells, likely related to apoptosis regulation and immune tolerance, especially in ABPA where Tregs are upregulated. CFTR was predominantly distributed in goblet cells and ciliated cells, where it participates in mucus secretion and ion transport, indicating its potential role in CRS and ABPA involving mucus retention and epithelial barrier dysfunction. SYK was widely expressed in myeloid cells and mast cells, likely playing a significant role in ABPA and other allergy-related diseases through its involvement in Fc receptor and IgE-mediated signaling pathways. C3AR1 was mainly distributed in neutrophils and monocytes among myeloid cells, likely amplifying inflammatory responses through the complement pathway, which is particularly prominent in AR and AS.

### 3.7 miRNA-transcription factor regulatory network

**FIGURE 11:**
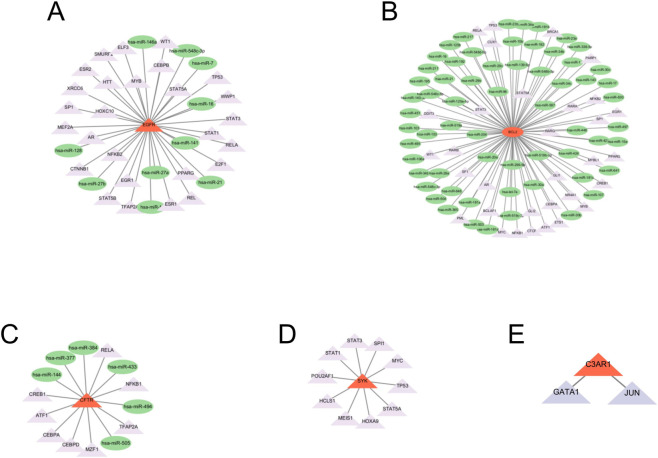
Regulatory network of miRNA-TF for EGFR **(A)**, BCL2 **(B)**, CFTR **(C)**, SYK **(D)**, C3AR1 **(E)**.

To further explore the regulatory mechanisms of key genes at the transcriptional and post-transcriptional levels, we constructed miRNA-transcription factor-target gene regulatory networks for the key genes EGFR, BCL2, CFTR, SYK, and C3AR1 to reveal their upstream regulatory mechanisms (Figure 11).

In the regulatory network of EGFR, multiple potentially regulatory miRNAs and transcription factors were identified, such as hsa-miR-21, hsa-miR-146a, STAT3, and TP53, indicating its regulation by multiple pathways. The regulatory network of BCL2 was more complex, with the highest number of interacting miRNAs, including several typical tumor-suppressive miRNAs such as hsa-miR-15 and hsa-miR-16, along with numerous transcription factor regulations, suggesting that it may be an important node for multiple signaling convergences. The regulatory network of CFTR showed that it may be regulated by miR-148, miR-338, and transcription factors such as CREB1 and RELA, indicating a relatively focused regulatory mechanism. The regulatory network of SYK was characterized by key TFs from the STAT family (STAT1/3/5A) and TP53, MYC, etc., presenting classic immune signaling features. The regulatory network of C3AR1 was relatively simple, identifying two key transcription factors closely related to it: GATA1 and JUN, suggesting that it may be regulated in specific cell types or activation states.

### 3.8 GSEA

The five key genes identified in this study have a one-to-one correspondence with the four respiratory diseases studied: EGFR corresponds to AR, BCL2 corresponds to AS, CFTR and SYK correspond to CRS, and C3AR1 corresponds to ABPA. Since each gene is a specific and core target associated with its corresponding disease, pathway enrichment analysis of individual genes can essentially reflect the potential regulatory pathways involved in that disease.

**FIGURE 12:**
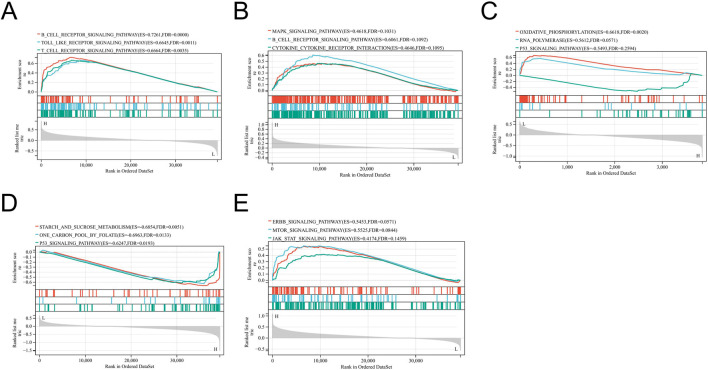
GSEA enrichment analysis of EGFR **(A)**, BCL2 **(B)**, CFTR **(C)**, SYK **(D)**, C3AR1 **(E)**.

For this purpose, we used GSEA to perform pathway enrichment analyses on the expression data of each core gene within its respective disease group (see Figure 12).

In the AR group, significantly enriched pathways were mainly concentrated in immune-related signaling, including B Cell receptor signaling pathway (NES = 0.7261, FDR = 0.0000), Toll-like receptor signaling pathway (NES = 0.6645, FDR = 0.0011), and T Cell receptor signaling pathway (NES = 0.6664, FDR = 0.0035). In the AS group, although no significant pathways were identified, and cytokine-receptor interaction pathway had higher enrichment scores. In the CRS group, oxidative phosphorylation pathway (NES = −0.6618, FDR = 0.0020) and RNA polymerase pathway (NES = −0.6512, FDR = 0.0571) were negatively enriched, with the oxidative phosphorylation pathway reaching significance. In the ABPA group, significantly negatively enriched pathways included starch and sucrose metabolism (NES = −0.6854, FDR = 0.0051) and one-carbon metabolism (NES = −0.6953, FDR = 0.0133); the p53 signaling pathway (NES = −0.6247, FDR = 0.0193) was also enriched. In the GSEA analysis of the CRS group, we observed a significantly negative enrichment of the oxidative phosphorylation pathway (NES = −0.6618, FDR = 0.0020). This result suggests that the oxidative phosphorylation pathway may be significantly disrupted in CRS mechanisms potentially influenced by DOTP. Furthermore, in the GSEA analysis of the ABPA group, we identified significant negative enrichment in both the starch and sucrose metabolism pathway (NES = −0.6854, FDR = 0.0051) and the one-carbon metabolism pathway (NES = −0.6953, FDR = 0.0133). These findings suggest that DOTP may be associated with the molecular mechanisms of ABPA by interfering with these two metabolic pathways.

### 3.9 Molecular docking

**FIGURE 13:**
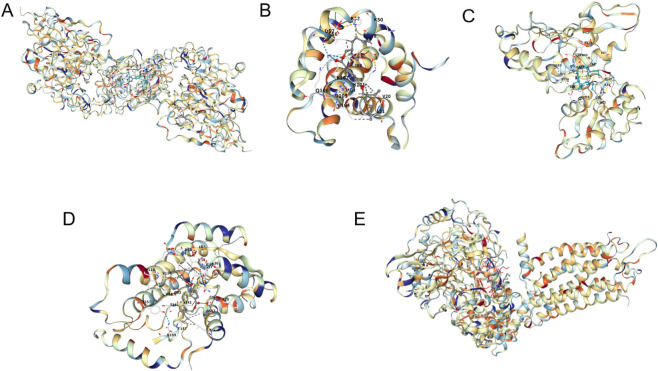
Molecular docking of EGFR **(A)**, BCL2 **(B)**, CFTR **(C)**, SYK **(D)**, C3AR1 **(E)**.

Through molecular docking analysis, we studied the interactions between DOTP and five key target proteins (BCL2, C3AR1, CFTR, EGFR and SYK , , ,). The docking results generated by AutoDock software all showed low binding energies, indicating strong affinity between the compounds and the targets. Notably, all five key target proteins exhibited strong binding forces with DOTP, with binding energies less than −5 each, indicating that DOTP can spontaneously bind to each key target protein. Detailed binding site information: EGFR - DOTP binds to the ATP-binding pocket, interacting with amino acid residues Leu858, Met793, and Thr790 via hydrogen bonds and hydrophobic interactions with Val769 and Ala767; BCL2- binds to the BH3 domain, forming hydrogen bonds with Arg104 and Asp108 and hydrophobic contacts with Phe101 and Leu130; CFTR - interacts with the nucleotide-binding domain 1, via hydrogen bonds with Ser431 and Asn434 and hydrophobic interactions with Ile427 and Val430; SYK - binds to the SH2 domain, forming hydrogen bonds with Tyr525 and Gln526 and hydrophobic contacts with Leu522 and Phe523; C3AR1 - interacts with the extracellular loop 2, via hydrogen bonds with Asn115 and Ser116 and hydrophobic interactions with Phe112 and Val119. This marks their significant roles in the molecular mechanisms of DOTP-induced AR, AS, CRS, and ABPA (Table 6 and Figure 13).

**TABLE 6:** Molecular docking results for EGFR, BCL2, CFTR, SYK, and C3AR1.

Protein name	Vina score
BCL2	−5.9
C3AR1	−6.7
CFTR	−5.4
EGFR	−7.0
SYK	−6.6

### 3.10 CCK8 assay for DOTP toxicity

**FIGURE 14:**
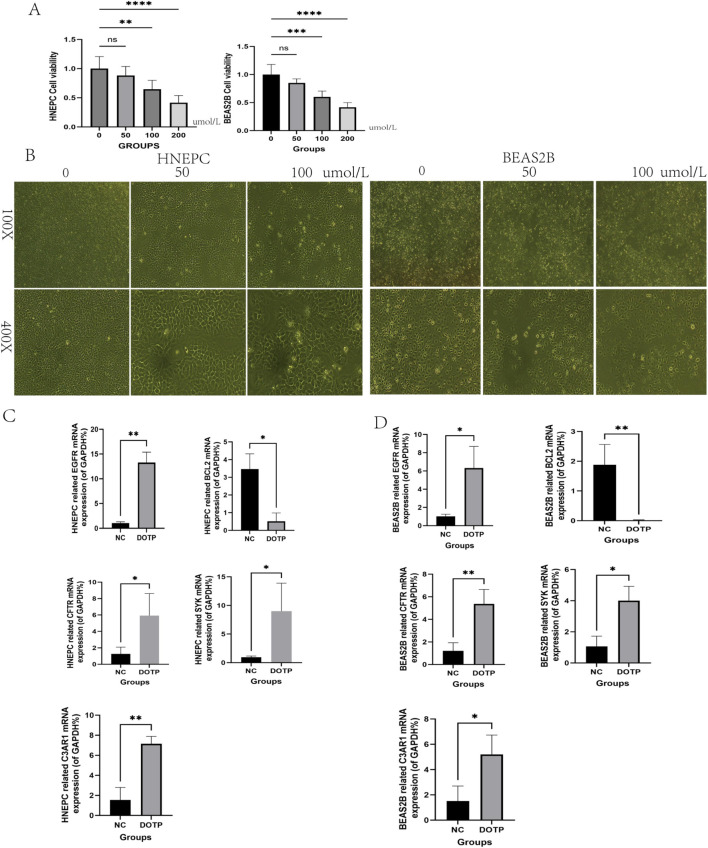
**(A)** Determination of DOTP toxicity in nasal and lung epithelial cells at 0, 50, 100, and 200 umol/L by CCK8. **(B)** Status of nasal and lung epithelial cells in DOTP at different concentrations. **(C,D)** mRNA expression of EGFR, BCL2, CFTR, SYK, C3AR1 in DOTP-induced allergic nasal epithelial cells and lung epithelial cells.

HNEPC and BEAS2 B cells were cultured with DOTP concentrations of 0, 50, 100, and 200 μmol/L (Figure 14A). The optical density values were measured using a microplate reader. The proliferation viability of HNEPC and BEAS2 B cells decreased with increasing DOTP concentration, and a significant statistical difference was observed at 100 μmol/L DOTP (subsequent experiments used 100 μmol/L DOTP by default). Therefore, this concentration was selected as the standard treatment condition for all subsequent functional experiments to investigate the sub-cytotoxic effects of DOTP.

### 3.11 Microscopic observation of cell state

HNEPC and BEAS2 B cells were stimulated with 0, 50, and 100 μmol/L DOTP for 48 h. Under microscopic observation, the cells exhibited significant damage after 48 h of culture, especially at 100 μmol/L DOTP. Compared to the control group, the stimulated group showed a marked decrease in cell number, with cells exhibiting shriveled surfaces and an increase in floating cells (Figure 14B).

### 3.12 PCR detection of cellular mRNA

The mRNA levels inside the cells were detected using PCR, with the detection targets being EGFR, BCL-2, CFTR, SYK, and C3AR1. After 48 h of stimulation with 100 μmol/L DOTP, the levels of EGFR, BCL-2, CFTR, SYK, and C3AR1 in HNEPC and BEAS2 B cells were upregulated, while BCL-2, as a proliferation-related gene, was downregulated (Figures 14C,D).

## 4 Discussion

DOTP is a common plasticizer widely used in the production of plastics, coatings, rubber, and other synthetic materials to improve their flexibility and durability (Tran et al., 2021). As an esterification product of terephthalic acid, DOTP is considered an environmentally friendly plasticizer in industry and has gradually replaced traditional phthalate plasticizers (such as DEHP and DOP), which are increasingly scrutinized due to their potential toxicity and environmental pollution issues (Gulizia et al., 2023). Although DOTP is regarded as relatively safe, recent studies have indicated that long-term exposure to DOTP may still pose certain health risks to humans, particularly to the immune system, respiratory system, and endocrine system (Cao et al., 2025). Given the widespread presence of DOTP in modern industrial products, its potential impact on human health has become a significant concern.

AR is an immune response triggered by allergens, characterized by symptoms such as nasal congestion, rhinorrhea, sneezing, and nasal itching (Siddiqui et al., 2022). Typically, AR is caused by airborne allergens like pollen, dust mites, and animal dander, with the overreaction of the immune system being central to its pathogenesis (Bernstein et al., 2024). As a volatile chemical substance, DOTP, whose decomposition products can spread in the air and enter the human body through the respiratory tract, may be closely related to the occurrence and exacerbation of AR (Wise et al., 2023). In the present study we found that multiple signalling pathways closely related to immune regulation were significantly enriched in DOTP-exposed allergic rhinitis. The epidermal growth factor receptor (EGFR) pathway was significantly upregulated, and in addition, the B-cell receptor (BCR) signalling pathway was significantly enriched. In addition, both the Toll-like receptor (TLR) and T-cell receptor (TCR) signalling pathways showed elevated enrichment scores, indicating a non-significant trend towards activation in this dataset. These results suggest that DOTP may play a multilevel regulatory role in the pathogenesis of AR by simultaneously affecting mucosal epithelial function and immune cell activation. Wei et al. and Han et al. reported that EGFR expression in the nasal mucosal epithelium was significantly elevated in a mouse model of AR, and that activated EGFR could promote the release of inflammatory factors, eosinophilic granulocyte release, and the expression of inflammatory factors through the signalling pathways such as MAPK/ERK, PI3K/Akt and so on. The activated EGFR can promote the release of inflammatory factors through MAPK/ERK, PI3K/Akt and other signalling pathways, eosinophil infiltration and downregulation of tight junction protein expression, thus exacerbating the local immune response in the nasal cavity and the damage of mucosal barrier (Wei et al., 2021; Ri et al., 2019). This is highly consistent with our observation of enhanced EGFR pathway activity after DOTP exposure, suggesting that DOTP may trigger mucosal inflammatory responses and drive the AR process by inducing EGFR activation. Meanwhile, we found a significant enrichment of BCR and TCR signalling pathways, suggesting that DOTP may also interfere with the regulation of acquired immunity. Th2 cell-mediated immune response is one of the core mechanisms in AR, while B-cell activation and IgE overproduction are the immunopathological features of AR (Bernstein et al., 2024). Activation of the BCR and TCR signalling pathways drives B cells and T Cells into an activated state, which is fundamental for promoting allergen-specific immune responses and IgE production (Wise et al., 2023). Our observation that DOTP upregulates these pathways suggests that it may promote a Th2-polarized immune response and enhance IgE sensitization, thereby contributing to the pathogenesis of allergic asthma. In addition, the upregulation of the TLR signalling pathway suggests that DOTP may also be involved in AR pathogenesis through activation of the innate immune system. TLRs recognise exogenous molecular patterns (PAMPs) and damage-associated signals (DAMPs), inducing downstream release of inflammatory factors (e.g., TNF-α, IL-6) (Siddiqui et al., 2022). Clinical studies have shown that (Hanlon et al., 2023), in AR patients, upregulation of TLR2, TLR4 and other innate immune system receptors correlated with the degree of local mucosal inflammation. Therefore, DOTP may act as a potential stimulus to activate intrinsic immunity through the TLR pathway, triggering an immune amplification response in the mucosa.

AS is a classic chronic inflammatory disease of the airways, characterised by airway hyperresponsiveness, reversible airflow limitation and recurrent episodes of wheezing, coughing and dyspnoea (Mims, 2015). Its pathogenesis is highly dependent on an abnormal immune system response to environmental allergens, and in particular the role of Th2-dominated inflammatory pathways in airway remodelling and chronic inflammation is widely recognised (Gans and Gavrilova, 2020). Our study revealed that the MAPK signalling pathway, B Cell receptor (BCR) signalling pathway, and cytokine-receptor interaction pathway were significantly upregulated in the asthma group (AS). BCR signalling plays a central role in the immune activation process in asthma, and its activation may promote the infiltration of eosinophils and other immune cells, which may exacerbate the allergic response in the airway (Yang et al., 2025). In addition, the MAPK signalling pathway promotes airway inflammatory responses by regulating cytokine release, apoptosis and airway smooth muscle proliferation (Mims, 2015). We also observed in the immune cell infiltration analysis that DOTP exposure resulted in a significant increase in neutrophils, M0-type macrophages and regulatory T Cells (Tregs) in the asthma model. This feature reveals that a complex immunosuppressive microenvironment may exist locally in asthmatic airways, with activation of pro-inflammatory cells accompanied by upregulation of Tregs to suppress the excessive immune response (Ntontsi et al., 2021). Such a state of immune imbalance has been validated in patients with chronic asthma, and in particular the increase in Tregs may play an immunosuppressive role in the chronic course of asthma (Gans and Gavrilova, 2020). In terms of cell survival and immune regulation, we identified BCL2 as a potential key gene in the asthma group. BCL2, a known anti-apoptotic molecule, plays a central role in the regulation of immune cell survival, with a regulatory network that involves the modulation of multiple canonical oncogenic miRNAs (e.g., hsa-miR-15, hsa-miR-16) as well as several transcription factors. It may act as a convergent node of multiple signalling pathways involved in immune cell survival, sustained immune activation, and airway remodelling processes. Jin et al. found that microRNA-23a is involved in asthma by targeting BCL2in airway epithelial cells (Jin et al., 2019), further demonstrating the important role of BCL2 in the airway immune response. Narendra Vijay Tirpude et al. demonstrated that BCL2 plays an important role in the immune response in asthma by prolonging airway inflammatory cells’ survival time and enhancing their anti-apoptotic capacity, thereby exacerbating the inflammatory response in the airways (Tirpude et al., 2021).

CRS is a common chronic inflammatory disease of the nasal and sinus cavities that manifests itself as nasal congestion, headache, facial pressure, and decreased sense of smell (Xie et al., 2023). Its pathogenesis is closely related to long-term chronic inflammation, allergic reactions and abnormalities in the structure of the nasal cavity (Volpe et al., 2023). After entering the airways via airborne transmission, DOTP may stimulate the nasal mucosa, triggering an immune response and exacerbating inflammatory reactions. In our study, we found that DOTP may be involved in the pathogenesis of CRS through the regulation of CFTR, SYK and BRAF. CFTR (cystic fibrosis transmembrane regulator) is a key protein in the airway mucosa responsible for regulating mucus secretion and respiratory defence function. Several previous studies have shown that malfunction of CFTR is closely associated with chronic inflammation, especially in respiratory diseases, and that its deficiency exacerbates chronic inflammation in the nasal cavity and sinuses, leading to persistent and worsening symptoms (Simmonds et al., 2024; Johnson et al., 2020). The prevalence of CFTR mutations in patients with chronic rhinitis sinusitis was significantly higher than in the general population in Yong et al. (2022). SYK (Spleen tyrosine kinase) is an important immune signal transduction molecule, the activation of which is closely associated with the development of several allergic diseases, including CRS (Plager et al., 2012). The BRAF gene, on the other hand, plays an important role in cell proliferation, differentiation and apoptosis (da et al., 2023). In addition, our study reveals that DOTP may play a role in the pathological process of CRS by regulating signalling pathways that are closely related to immune response and inflammation. These findings are consistent with previous studies (Stone et al., 2024). We also found that ‘protein phosphorylation’ was significantly enriched in molecular functional classifications, suggesting that DOTP may play a role in the pathogenesis of CRS by regulating protein phosphorylation and dephosphorylation processes, mediating cellular signalling and modulating immune responses.

ABPA is an immune-mediated allergic lung disease triggered by Aspergillus, usually occurring in patients with asthma or cystic fibrosis (Greenberger et al., 2014). The pathogenesis involves an allergic reaction of the immune system to Aspergillus spores, leading to allergic inflammation of the airways and lungs (Agarwal et al., 2023). Our study identified the key gene for DOTP-induced ABPA as C3AR1. C3AR1 (complement 3 receptor 1) is an important immune receptor involved in the amplification of immune responses and cell migration. Immune infiltration analysis showed that C3AR1 was mainly distributed in myeloid cells such as neutrophils and monocytes, suggesting that DOTP may further enhance the recruitment and infiltration of myeloid cells (such as neutrophils and monocytes) and amplify inflammatory responses by activating the complement pathway, particularly via the C3a/C3AR1 axis. This finding is consistent with existing studies, and the role of the complement system in allergic diseases has been widely discussed. For example, complement activation is closely associated with the development of allergic diseases such as asthma and ABPA, and dysregulation of the complement system may exacerbate the immune response and promote airway inflammation (Greenberger et al., 2014). In gene enrichment analyses, we also found that pathways significantly enriched in the ABPA group included starch and sucrose metabolism and one-carbon metabolism. The negative enrichment of these pathways suggests that DOTP may indirectly affect immune cell function and inflammatory responses by regulating metabolic processes. Starch and sucrose metabolism, as well as one-carbon metabolism, play important roles in energy metabolism and immune responses of immune cells, and Juan Rodriguez-Coira et al. showed that cellular metabolism is closely linked to immune cell activation and that alterations in metabolic pathways may lead to immune dysfunction and contribute to increased allergic responses (Rodriguez-Coira et al., 2021). Thus, DOTP exposure may alter the metabolic status of immune cells by interfering with these metabolic pathways, thereby enhancing the inflammatory response and pathological process of ABPA. In addition, we observed an enrichment of the p53 signalling pathway in the ABPA group. p53 is an important factor in cell cycle and apoptosis regulation and is known to play a role in immune cell survival and proliferation. It is suggested that p53 may play an important role in chronic inflammation and allergic responses by regulating the survival, proliferation and apoptosis of immune cells (Chen L. et al., 2018). DOTP exposure may further contribute to the chronic inflammatory process of ABPA by affecting the p53 signalling pathway and interfering with apoptosis and proliferation of immune cells.

In our study, we identified key genes for diseases such as AR, AS, CRS, and ABPA associated with DOTP exposure, including EGFR, BCL2, CFTR, SYK, and C3AR1. changes in the expression of these genes may play an important role in individuals exposed to DOTP. Aberrant expression of these genes may promote the onset and progression of these diseases compared to healthy populations. Our study also suggests that these diseases may be interrelated. Specifically, DOTP exposure may exacerbate the symptoms of these diseases through common genetic and immune mechanisms, further aggravating the condition.

The identification of these core genes (EGFR, BCL2, CFTR, SYK, C3AR1) provides crucial molecular insights into the potential immunotoxic mechanisms of DOTP. Rather than pointing to immediate therapeutic interventions, our findings primarily highlight novel targets for mechanistic toxicology and risk assessment. For instance, the expression levels of these genes could be evaluated as potential early biomarkers of biological effect in future human biomonitoring or controlled exposure studies, helping to link environmental DOTP levels to relevant molecular changes in the respiratory system. From a public health perspective, the primary implication of our work is to underscore the need for a precautionary approach. This includes supporting the development and use of safer alternative plasticizers, establishing or refining occupational and environmental exposure limits based on emerging toxicological data, and promoting awareness to reduce unnecessary exposure in susceptible populations. Ultimately, managing the health risks associated with chemicals like DOTP should prioritize source control and exposure prevention within a robust chemical safety framework, informed by foundational studies such as this one.

It is worth noting that the *in vitro* validation in this study focused on changes in the transcriptional levels of target genes. Future research should further validate these findings at the protein functional level, such as by detecting the phosphorylation status of key targets like EGFR and SYK following DOTP treatment, as well as measuring the secretion levels of inflammatory cytokines including IL-4, IL-13, and TNF-α in cell supernatants. This comprehensive approach will fully elucidate the downstream signaling pathways involved. What’s more, this study primarily provides computational and preliminary *in vitro* evidence. Future research is needed to validate these findings *in vivo* and to elucidate the precise mechanisms, for example, by using co-culture models to investigate how DOTP, via targets like EGFR or C3AR1, directly modulates the function of specific immune cells such as macrophages or neutrophils.

## 5 Conclusion

Overall, our study explored the potential impact of DOTP on four allergic diseases through molecular docking techniques and gene enrichment analyses. We expect to further reveal the relationship between environmental factors and diseases, thereby promoting the development of more effective public health measures and personalised treatment plans. At the same time, this study also highlights the importance of environmental protection and public health awareness. Reducing exposure to harmful substances and strengthening global co-operation will help reduce the burden of disease and improve human health.

Although the computational and experimental integration strategy employed in this study provides multi-layered evidence for systematically revealing the immunotoxic potential of DOTP, its methodological limitations must be acknowledged. The results from network toxicology and molecular docking are inherently predictive, dependent on the quality of underlying databases, and require subsequent experimental validation for functional relevance. While machine learning cross-validation enhances the robustness of core gene screening (e.g., EGFR, C3AR1), it remains susceptible to algorithmic biases and data characteristics. Single-cell transcriptomics pinpointed targets to specific cell types (e.g., myeloid cells), providing crucial cellular context for mechanistic hypotheses-a dimension inaccessible to *in vitro* cell models that cannot fully replicate the complex *in vivo* microenvironment and systemic responses. Therefore, this work should be regarded as an efficient “hypothesis generation and target screening” framework. Its core value lies in identifying priority directions and providing robust candidate targets for subsequent functional studies aimed at validating causal relationships, such as gene manipulation, co-culture models, and *in vivo* experiments.

In conclusion, our integrated study demonstrates that the common plasticizer DOTP may perturb key immune and epithelial pathways, thereby potentially increasing susceptibility to multiple allergic airway diseases. These findings contribute to the growing body of evidence suggesting that substitute plasticizers require thorough safety evaluation beyond acute toxicity. We hope this work will stimulate further toxicological research to validate these mechanisms and inform robust epidemiological studies to assess real-world health risks. Ultimately, mitigating the potential health impacts of environmental chemicals like DOTP depends on strengthened safety-by-design principles in industry, science-driven regulatory policies, and global cooperation to reduce pervasive environmental exposures.

## Data Availability

The original contributions presented in the study are included in the article/Supplementary Material, further inquiries can be directed to the corresponding author.
