# Supplementary material for: Deciphering the toxicological mechanism of airway allergic diseases caused by dioctyl terephthalate: a composite study
Source: Front Toxicol. 2026 Mar 11;8:1743420. doi: 10.3389/ftox.2026.1743420 (PMC13012849; doi:10.3389/ftox.2026.1743420)
Supplement: Supplementary file 1 [file Presentation1.pptx]

## Slide 1
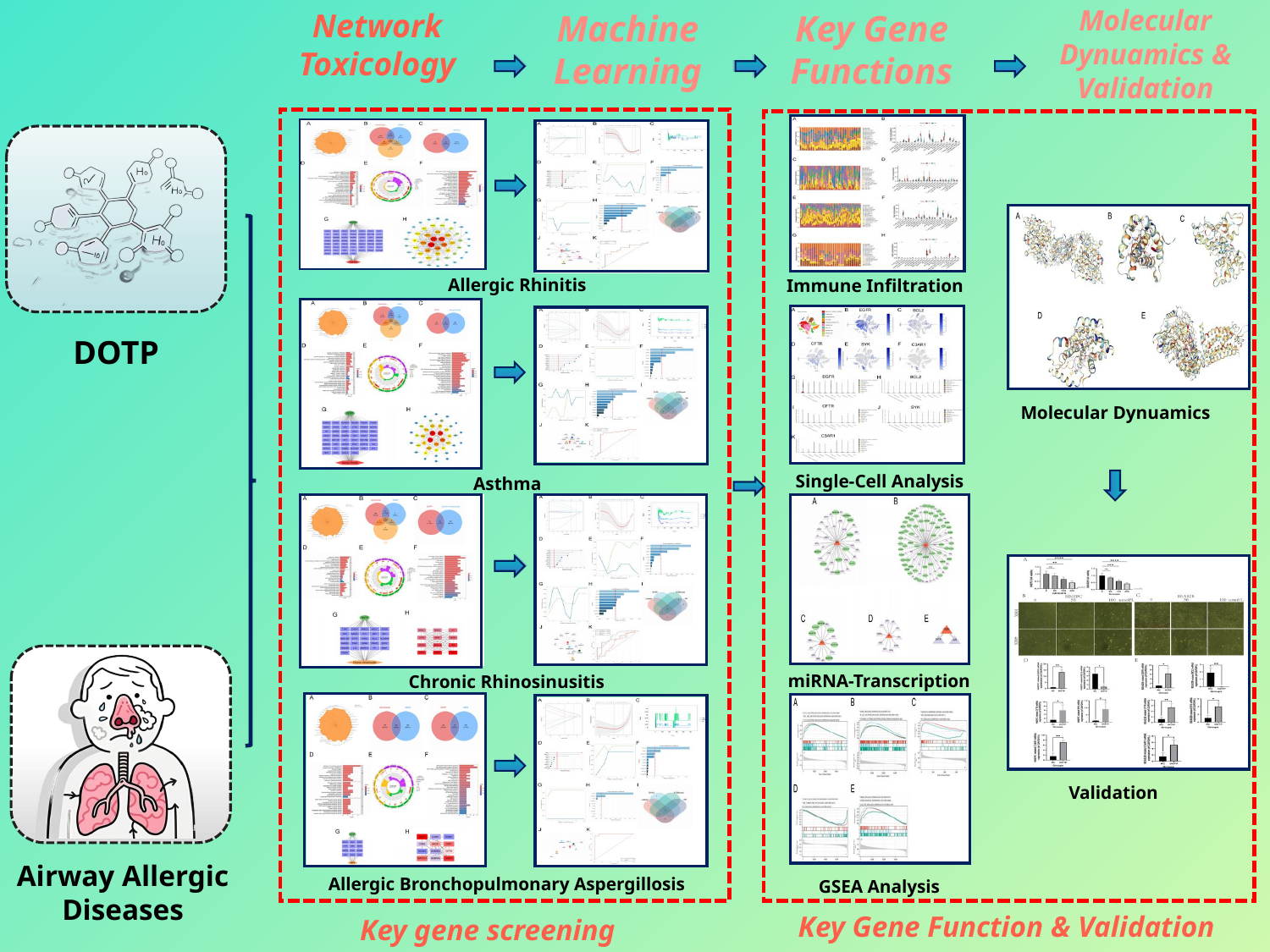

Network Toxicology
Machine Learning
Key Gene Functions
Molecular Dynuamics & Validation
-
-
-
Allergic Rhinitis
Immune Infiltration
DOTP
Molecular Dynuamics
Single-Cell Analysis
Asthma
miRNA-Transcription
Chronic Rhinosinusitis
Validation
Airway Allergic Diseases
Allergic Bronchopulmonary Aspergillosis
GSEA Analysis
Key Gene Function & Validation
Key gene screening
